# A New Tattooing Apparatus

**Published:** 1898-06

**Authors:** 


A New Tattooing Apparatus.—Otto Bertram, of Vienna, has invented a new tattooing instrument. The needles of the apparatus leave a very fine hole, and pass each time through a layer of coloring matter, so that every mark is indelibly imprinted. In swine this stamp is clear and plain even after scalding. The price is about ten dollars. Further information may be had at 73 Frankfurter Allée, Vienna. Any monogram can be made.—*Idem.*

